# 

**Published:** 1856-04

**Authors:** 


COMMUNICATIONS FOR PUBLICATION TO BE ADDRESSED TO 'EDITORIAL COMMITTEE.'

### IOWA MEDICAL JOURNAL.

CONDUCTED BY THE FACULTY OF THE

### COLLEGE OF PHYSICIANS AND SURGEONS

OF THE

### IOWA UNIVERSITY.

VOL. III. MARCH AND APRIL, 1856. NO. 2.

PUBLISHED EVERY ALTERNATE MONTH.

TERMS—TWO DOLLARS IN ADVANCE.

EDITORIAL COMMITTEE,

D. L. M'GUGIN, M. D.

JNO. R. ALLEN, M. D.

FINANCE COMMITTEE.

J. C. HUGHES, M. D.

KEOKUK, IOWA.

PRINTED AT THE POST BOOK AND JOB OFFICE.

BUSINESS COMMUNICATIONS TO THE 'FINANCE COMMITTEE.'

